# Arctigenin inhibits STAT3 and exhibits anticancer potential in human triple-negative breast cancer therapy

**DOI:** 10.18632/oncotarget.13393

**Published:** 2016-11-16

**Authors:** Tingting Feng, Wei Cao, Wanxiang Shen, Liang Zhang, Xinsheng Gu, Yang Guo, Hsiang-i Tsai, Xuewen Liu, Jian Li, Jingxuan Zhang, Shan Li, Fuyun Wu, Ying Liu

**Affiliations:** ^1^ Laboratory of Molecular Target Therapy of Cancer, Institute of Basic Medical Sciences, Hubei University of Medicine, Shiyan, Hubei 442000, China; ^2^ School of Basic Medical Sciences, Anhui Medical University, Hefei, Anhui 230032, China; ^3^ MOE Key Laboratory of Industrial Fermentation Microbiology, College of Biotechnology, Tianjin University of Science & Technology, Tianjin 300457, China; ^4^ Graduate School at Shenzhen, Tsinghua University, Shenzhen, Guangdong 518055, China; ^5^ School of Basic Medical Sciences, Hubei University of Medicine, Shiyan, Hubei 442000, China

**Keywords:** arctigenin, STAT3, triple-negative breast cancers, computational docking, taxotere

## Abstract

Triple-negative breast cancers (TNBCs) are the most aggressive and hard-to-treat breast tumors with poor prognosis, and exploration for novel therapeutic drugs is impending. Arctigenin (Atn), a bioactive lignan isolated from seeds of *Arctium lappa* L, has been reported to inhibit many cancer types; however, the effect of Atn on TNBC remains unclear. In this study, we demonstrated that Atn decreased proliferation, and induced apoptosis in TNBC cells. Furthermore, we explored the underlying mechanism of Atn inhibition on TNBC cells. Computational docking and affinity assay showed that Atn bound to the SH2 domain of STAT3. Atn inhibited STAT3 binding to genomic DNA by disrupting hydrogen bond linking between DNA and STAT3. In addition, Atn augmented Taxotere^®^-induced TNBC cell cytotoxicity. TNBC xenograft tests also confirmed the antitumor effect of Atn *in vivo*. These characteristics render Atn as a promising candidate drug for further development and for designing new effective STAT3 inhibitors.

## INTRODUCTION

Breast cancer is one of the most prevalent carcinoma and the second leading cause of mortality in women worldwide; more than one million cases of breast cancer occur, and more than 400,000 deaths are recorded annually worldwide [1]. Approximately, 12%-24% of breast cancers are triple-negative breast cancer (TNBC) [2]. TNBC is characterized by the absence of estrogen receptor, progesterone receptor, and HER-2 expression; this cancer results in high morbidity and mortality because of its rapid growth rate, metastatic potential, and frequently acquired treatment resistance [3]. Studies have shown that tumors of this aggressive type are of higher histological grade, afflict a great number of young women, and are more likely to recur earlier at distant sites, thereby leading to poor overall prognosis [4, 5]. TNBC is the only major type of breast cancer without specific targeted therapy for improving patient outcomes. Novel agents that can inhibit the proliferation and metastasis of TNBC cells must be identified to improve treatment outcomes in patients.

Signal transducers and activators of transcription (STATs) are a family of transcription factors that can be activated by tyrosine phosphorylation by the members of the Jak tyrosine kinase family in response to a variety of cytokines and growth factors [6, 7]. This family, including STATs 1, 2, 3, 4, 5a, 5b, and 6, promote tumor cell survival, proliferation and drug resistance [8]. In cancer, STATs are often constitutively activated in response to the abnormal binding of a large number of hormones and cytokines to their corresponding receptors [9]. Among the STATs, STAT3 is often constitutively actived in various human cancers including mammary of solid carcinoma and hematologic malignancies [10]. Especially in TNBC, a high percentage of carcinomatous tissues exhibit persistent STAT3 activation [11]. Previous studies found that a majority of STAT3 abnormal activation derived from upstream signaling dysregulations, which over-phosphorylated STAT3 [11, 12]. Whereas, only a fraction of STAT3 over-activation results from somatic mutations [13]. Activated STAT3 can up-regulate genes encoding cell-cycle regulators (Cyclin D1 and c-Myc), apoptosis inhibitors (Mcl-1, Bcl-xl, and survivin), and angiogenesis inducers (VEGF). These genes are important molecular cubs for tumor progression [8, 14]. In addition, aberrant STAT3 expression is significantly associated with cell survival, epithelial to mesenchymal transition (EMT), lymphoma invasion, and metastasis by regulating these pivotal intracellular signaling cascades in various malignant diseases [15–18]. STAT3 is important for breast involution after weaning and considered a prognostic factor for breast cancer [19].

**Figure 1:**
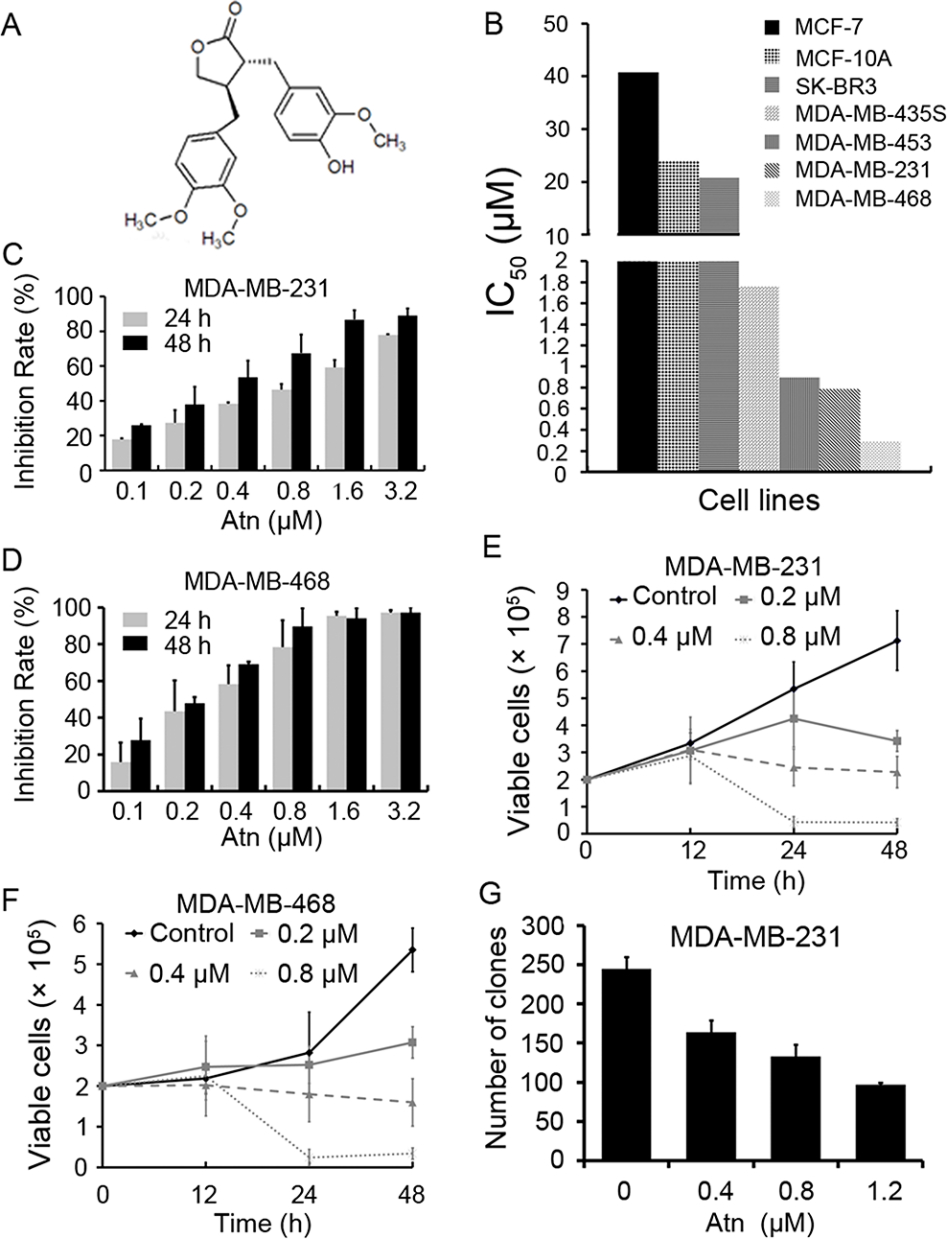
Atn decreased viability of breast cancer cells (**A**) Chemical structure of Atn. (**B**) The IC_50_ of Atn for indicated cell lines. (**C** and **D**): The inhibitory effects of Atn on MDA-MB-231 and MDA-MB-468 cells analyzed by MTT assay. (**E** and **F**) Inhibitory effects of Atn on cell viability of MDA-MB-231 and MDA-MB-468 cells assayed by trypan blue exclusion assay. (**G**) The colony formation assays of MDA-MB-231 cells treated with Atn at indicated concentration.

*Arctium lappa* L. (Asteraceae) is a medicinal plant widely distributed in China, Japan, and Korea. This plant is used in folk medicine as diuretic, anti-inflammation, anti-hypertension, depurative, anti-ulceration, digestive stimulant, and antioxidant agents [20, 21]. Arctigenin (Atn, Figure 1A) is a bioactive lignan isolated from the seeds of A. lappa L. Atn exerts anti-inflammatory effects by inhibiting nuclear transcription factor-kappa B (NF-κB) [19]. Atn can also enhance the chemosensitivity of several cancer cells (HepG2, HeLa, and K562) to cisplatin at high doses [22].

The mechanism through which Atn inhibits the proliferation of human TNBC cells or enhances the chemosensitivity of cancer cells remains less understood. In this study, we investigated the effect of Atn on human breast cancer cells and the mechanisms of Atn against highly invasive TNBCs.

## RESULTS

### Effects of Atn on cell proliferation and growth of breast cancer cell lines

We tested the effects of Atn on breast cancer and normal human mammary epithelial cell lines. The normal human mammary epithelial cell line MCF-10A was less sensitive to Atn than MDA-MB-435S, MDA-MB-453, MDA-MB-231, and MDA-MB-468 breast cancer cell lines (Figure 1B). Poor migratory cell lines, MCF-7 and SK-BR3 cells, were the most resistant to Atn with IC_50_ > 20 μM for 24 h (Figure 1B). Interestingly, highly invasive (MDA-MB-231 and MDA-MB-468) TNBC cells were more sensitive to Atn with IC_50_ of 0.787 and 0.283 μM, respectively (Figure 1B–1D; Table 1). Atn inhibited the growth of MDA-MB-231 (Figure 1E) and MDA-MB-468 (Figure 1F) cells in a time- and dose-dependent manner, as assessed by trypan blue exclusion assay. Atn also significantly suppressed the clonogenic activity of MDA-MB-231 cells (Figure 1G). Therefore, highly invasive TNBC cell lines, MDA-MB-231 and MDA-MB-468, are more sensitive to Atn treatment than the other cell lines. Basing on these results, we conducted the following experiments to investigate the mechanism of Atn in TNBC cells by using MDA-MB-231 and MDA-MB-468 cells.

**Table 1:** IC_50_s of Atn on breast cancer cell lines

Cell lines	MCF-7	MCF-10A	SK-BR3	MDA-MB-435S	MDA-MB-453	MDA-MB-231	MDA-MB-468
IC50 (μM)	40.81 ± 3.43	24.10 ± 4.07	20.67 ± 2.71	3.76 ± 0.59	2.90 ± 0.52	0.79 ± 0.06	0.29 ± 0.04

The cells were treated with Atn at various concentrations for 24 h, the cell cytotoxicity was analyzed by MTT assay, and the IC_50_ was calculated using CalcuSyn (version 2.0, Biosoft, Cambridge, UK). Values shown are means plus or minus SD of quadruplicate determinations.

### Atn inhibits invasion, migration and induces apoptosis of TNBC cells

**Figure 2:**
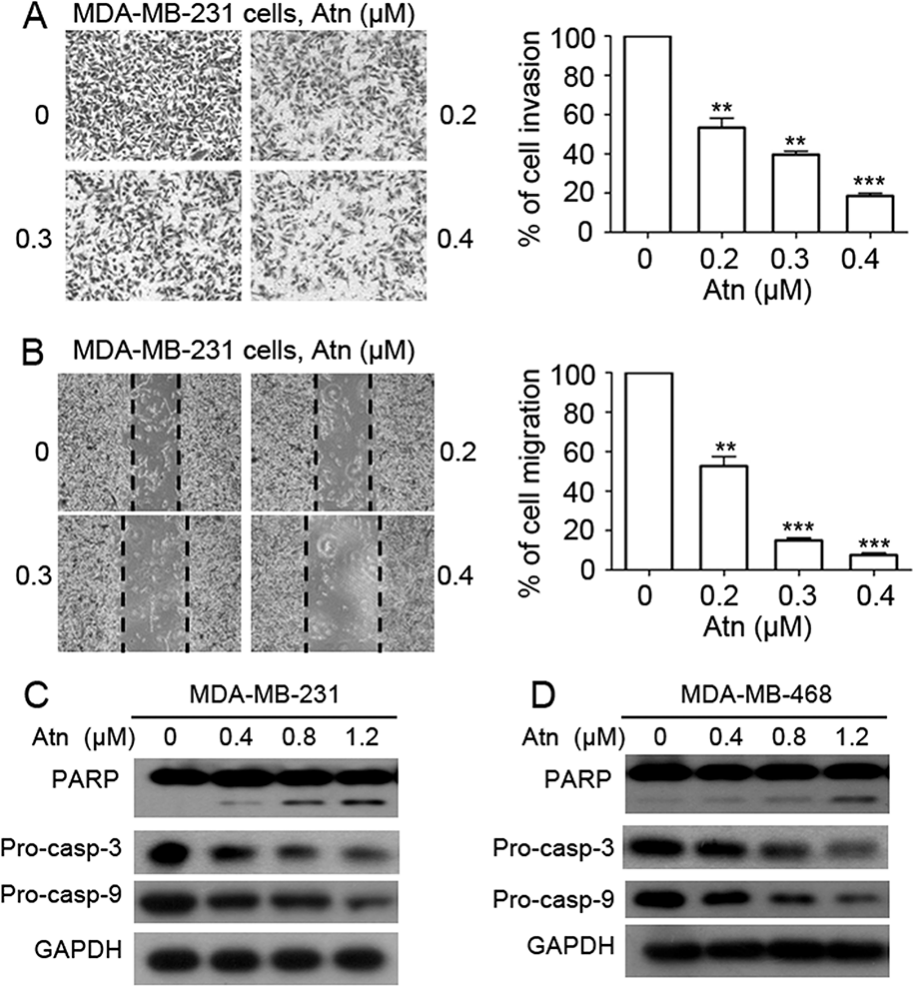
Atn reduces invasive behavior and induces apoptosis of TNBC cells (**A**) Invasion assay was carried out using modified 24-well microchemotaxis chambers. Then randomly chosen fields were photographed (×100), and the number of cells migrated to the lower surface was calculated as a percentage of invasion. Data are shown as the mean ± SD of three independent experiments by analysis of Student's *t* test. *P < 0.05, **P < 0.01, and ***P < 0.001, vs 0 μM. (**B**) Confluent cells were scratched and then treated with Atn in a complete medium for 24 h. The number of cells migrated into the scratched area was photographed (×40) and calculated as a percentage of migration. Data are shown as the mean ± SD of three independent experiments by analysis of Student's *t* test. *P < 0.05, **P < 0.01, and ***P < 0.001, vs 0 μM. (**C–D**) MDA-MB-231 and MDA-MB-468 cells were treated with Atn at the indicated concentrations for 24 h, followed by Western blot analysis for PARP, pro-caspase-3 (pro-casp-3), and pro-caspase-9 (pro-casp-9). GAPDH was used as a loading control.

Considering that highly invasive TNBCs are more sensitive to Atn treatment than poorly invasive cells, we determined whether Atn inhibits the invasion and migration of breast cancer cells. Invasion assay was performed in MDA-MB-231 cells by using Matrigel-coated 24-well microchemotaxis chambers in the presence of Atn. Atn (0–0.4 μM) suppressed the invasion of MDA-MB-231 cells (Figure 2A). To further explore the effect of Atn on cell migration, we treated MDA-MB-231 cells with Atn (0–0.4 μM) and performed wound healing assay after 24 h. Atn significantly reduced MDA-MB-231 cell migration in a dose-dependent manner (Figure 2B). These results suggested that Atn exhibited anti-invasive behavior toward TNBCs at low-cytotoxic concentrations.

To determine the cell death pathways induced by Atn, we treated MDA-MB-231 and MDA-MB-468 cells with increasing Atn concentrations for 24 h. Western blot results showed the presence of PARP cleavage and a significant dose-dependent decrease in pro-caspases 3 and 9 in Atn-treated cells (Figures 2C and 2D). These results indicated that Atn induced TNBC cell death via caspase-dependent apoptosis.

### Atn inhibits STAT3 (Y705) activation in human TNBC cells

**Figure 3:**
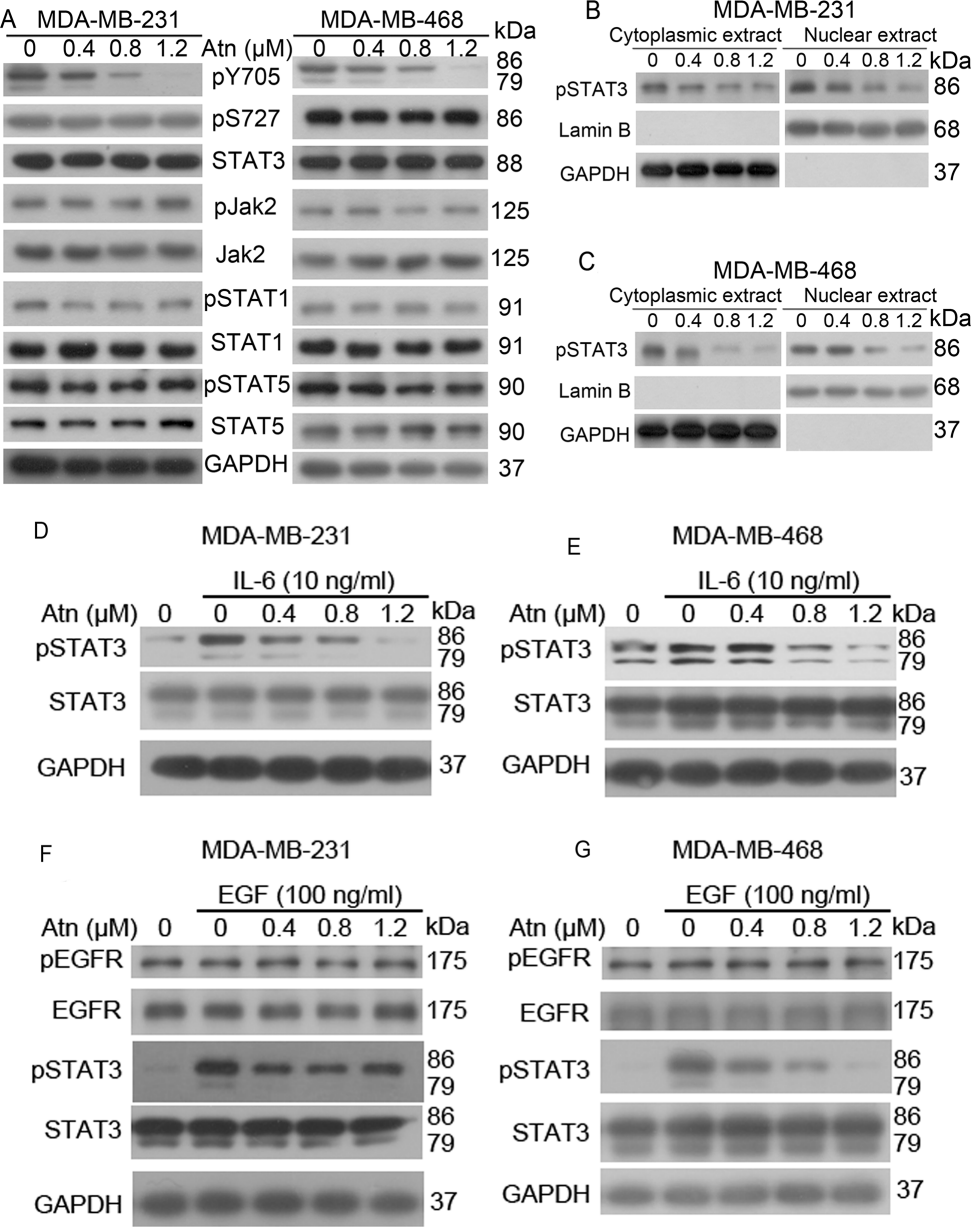
Atn inhibits STAT3 (Y705) activation in human TNBC cells (**A**) MDA-MB-231 and MDA-MB-468 cells were treated with Atn at the indicated concentrations for 24 h, followed by Western blot analysis for indicated antibodies. GAPDH was used as a loading control. (**B–C**) Cytoplasmic and nuclear fractions of MDA-MB-231 and MDA-MB-468 cells were isolated, the concentrations of nuclear and cytoplasmic protein were measured by Bradford assay, the same amount of nuclear and cytoplasmic protein was subjected to SDS gel, and Western blot were performed with anti-pSTAT3, GAPDH and Lamin B antibodies. (**D–G**) MDA-MB-231 and MDA-MB-468 cells were pretreated with the indicated concentrations of Atn for 24 h and stimulated with IL-6 for 30 min and with EGF for 10 min. Whole cell lysates were subjected to Western blot to determine the pSTAT3 (Tyr705), STAT3, pEGFR, and EGFR protein levels.

Human breast cancer cells express constitutively active STAT3 [3]. Whether Atn can inhibit the constitutive STAT3 activation in TNBC cells was examined. MDA-MB-231 and MDA-MB-468 cells were treated with increasing Atn concentrations for 24 h and subjected to Western blot analysis to assess signaling changes. Atn significantly reduced the tyrosine phosphorylation of STAT3 (p-STAT3 at Tyr705) and did not alter STAT3 protein expression. We also assessed the effect of Atn on pSer727, which was constitutively phosphorylated in both MDA-MB-231 and MDA-MB-468 cell lines. Atn did not reduce pS727 in both cell lines (Figure 3A). All seven STAT family members are detectable, specifically STAT 1, 3, and 5a/b, in all breast cancer subtypes [23]. Whether Atn inhibits the activation of other STAT proteins in TNBCs was further investigated. Under the condition that Atn completely inhibits STAT3 phosphorylation, neither the level of constitutively phosphorylated STAT5 and STAT1 nor the expression of STAT5 and STAT1 proteins was altered (Figure 3A). Phosphorylation of STAT3 leads to its dimerization and translocation from the cytoplasm into the nucleus. To confirm whether Atn inhibits the nuclear translocation of STAT3, we prepared nuclear and cytoplasmic extracts of Atn-treated and untreated cells by using NE-PER Nuclear and Cytoplasmic Extraction Kit. Exposure to Atn substantially inhibited the translocation of STAT3 from the cytoplasm to the nucleus (Figures 3B and 3C).

IL-6-stimulated STAT3 phosphorylation is a common model for studying STAT3 inhibition [24]. In clinic, IL-6-mediated STAT3 abnormal activation predicts tumor progression and poor prognosis in breast, lung, and hematopoietic tumors with gp130 constitutive activation [25] or elevated IL-6 levels [26, 27]. Hence, we examined whether Atn inhibits IL-6-stimulated STAT3 phosphorylation. IL-6 (10 ng/ml) treatment induced STAT3 activation (Figures 3D and 3E). However, pretreatment with Atn significantly inhibited IL-6-induced STAT3 activation. This pretreatment also decreased STAT3 activation to levels below that in non-stimulated cells. EGF activated STAT3 via gp130-independent EGFR [28] and a strong correlation was found between nuclear STAT3 activation and EGFR expression in breast cancers through immunohistochemical analysis [29]. Moreover, somatic-activating mutations in EGFR resulted in STAT3 over-activation through IL-6 production in human lung adenocarcinomas [30]. Therefore, we examined whether Atn inhibits EGF-stimulated STAT3 phosphorylation. EGF (100 ng/ml) treatment resulted in EGFR and STAT3 activation (Figures 3F and 3G). Nevertheless, pretreatment with Atn significantly inhibited EGF-induced STAT3 activation, but Atn treatment did not alter EGFR phosphorylation. These results suggested that Atn can inhibit IL-6 and EGF-mediated STAT3 abnormal activation.

### Atn is a STAT3 inhibitor

**Figure 4:**
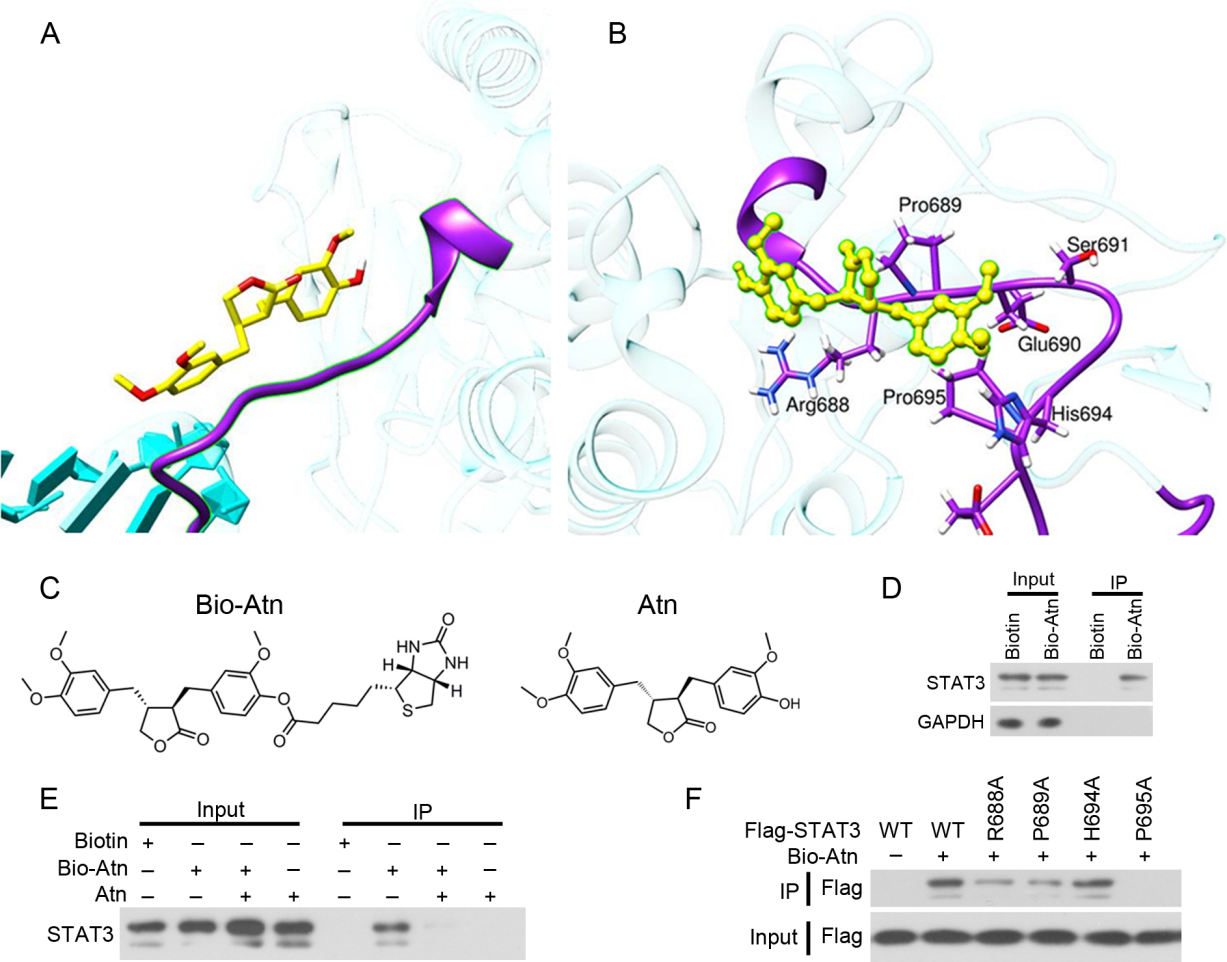
Atn directly binds STAT3 (**A**) Computational approaches to examine the binding of Atn and pSTAT3 in silico. Atn is shown as a yellow stick model, the SH2 domain of STAT3-Y705-DNA complex is shown in purple ribbon. (**B**) Details of the binding site of Atn in the STAT3 SH2 domain. The protein backbone is portrayed as a transparent sky blue ribbon; the main residues involved in drug interactions are shown as labeled purple sticks. Atn is portrayed as atom-yellowed sticks-and-balls. (**C**) Chemical structure of Bio-Atn and Atn. (**D**) MDA-MB-231 cells were treated with 10 μM Biotin or Bio-Atn for 12 h, lysed, and the cell lysates were subjected to immunoprecipitation using S. agarose and Western blot using indicated antibodies. (**E**) MDA-MB-231 cells were treated with Bio-Atn (5 μM) in the presence or absence of Atn (25 μM) for 3 h, lysed, and the cell lysates were subjected to immunoprecipitation and Western blot. (**F**) 293T cells were transfected with wild type (WT) or mutant STAT3 (R688A, P689A, H694A, or P695A) for 24 h, lysed, the lysates were subjected to immunoprecipitation using S. agarose and Western blot using indicated antibodies.

Various computational approaches were used to examine the binding between Atn and pSTAT3 *in silico*. Atn was computationally docked onto the SH2, linker, DNA-binding, and coiled-coil domains; the relevant Atn/protein affinities were scored by Autodock 4.2 program and molecular dynamics simulation (MDS). Considering the free energy values of binding G-bind and entropic components predicted for the interaction of Atn with the STAT3 domains obtained, we ascertained that Atn binds to the pSTAT3 SH2 domain and induces the inhibition of STAT3-Y705 phosphorylation. To understand the basis of the remarkable high affinity of Atn for pSTAT3, we performed a per-residue decomposition analysis of the free energy of binding. The interaction spectrum showed that the residues mostly involved in the binding to Atn clustered in the region ranging from Arg688 to Pro695 in the SH2 domain (Figure 4A). Notably, four residues (Arg688, Pro689, His694 and Pro695) were engaged in major stabilizing interactions with Atn (Figure 4B).

To experimentally confirm Atn/STAT3 interaction, biotinylated Atn (Bio-Atn, Figure 4C) was synthesized and validated by nuclear magnetic resonance (Supplementary Figure S1), with a purity of 95% determined by HPLC. In Bio-Atn-treated MDA-MB-231 cells, STAT3 was pulled down by streptavidin agarose (S. agarose, Figure 4D). The *in vitro* experiment showed that the binding of Bio-Atn to STAT3 was significantly attenuated by unlabeled Atn (Figure 4E), indicating the direct binding of Atn to STAT3. To confirm which residues were critical for the Atn interaction, site-directed mutagenesis on STAT3 was performed and plasmids containing wild type (WT) or mutant STAT3 (R688A, P689A, H694A, or P695A) were transfected into MDA-MB-231 cells to purify STAT3 protein for *in vitro* binding analysis (Figure 4F). The results showed that WT STAT3 strongly recruited Atn, while H694A mutation has no competitive inhibition to the binding affinity of Atn/STAT3; however, R688A, P689A, and P695A mutations drastically inhibited STAT3 binding to Atn (Figure 4F), indicating that R688, P689, and P695, especially P695 of STAT3 is critical for Atn-STAT3 binding.

### Atn inhibits STAT3 downstream target genes expressions via inhibition of STAT3 binding to genomic DNA

**Figure 5:**
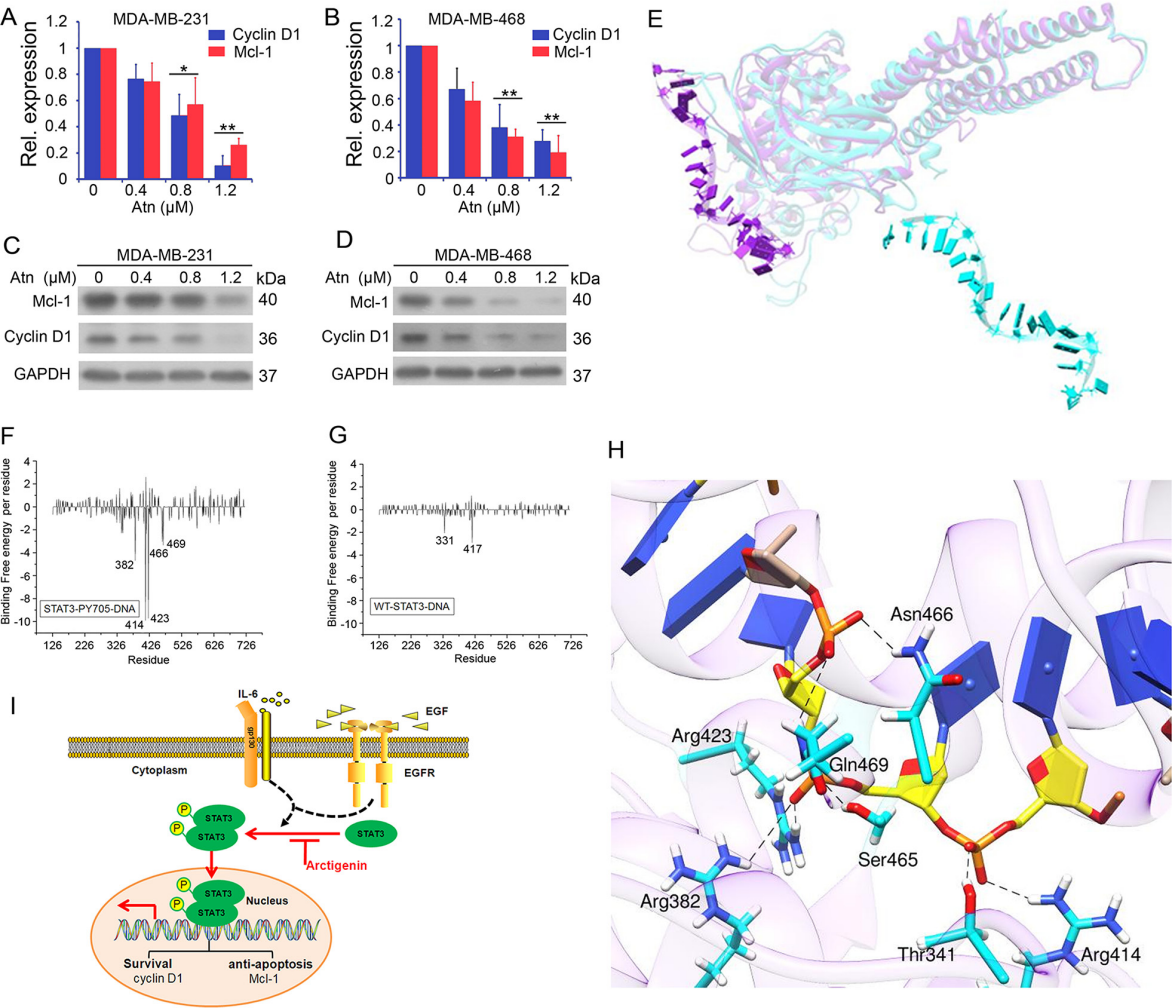
Atn inhibits STAT3 downstream target genes expression by inhibition of STAT3 binding to genomic DNA (**A–B**) cyclin D1 and Mcl-1 gene expression was detected by Real-time PCR analysis. GAPDH was used here as a housekeeping gene. (**C–D**) cyclin D1 and Mcl-1 protein expression was detected by Western blot. GAPDH was used here as a loading control. (**E**) Superposition of the average structure of STAT3-WT-DNA and STAT3-Y705-DNA from the last 50 ns molecular simulation trajectories. In the superimpositions, the protein residues and nucleic acids are shown in Ribbon. STAT3-WT protein is colored in cyan, STAT3-Y705 protein in purple. (**F–G**) Decomposition of binding free energy on per-residue basis for each complex. (**H**) Average complex structures of STAT3-Y705-DNA from the last 50 ns equilibrium trajectory, the DNA in STAT3-Y705-DNA complex is shown as a yellow stick model, hydrogen bonds are shown as dashed lines, the catalytic residues in STAT3-Y705-DNA complex is shown in cyan stick model. (**I**) Diagram of Atn blockage possible mechanism in TNBC cells.

To validate the effect of Atn on the transcription activity of STAT3, we detected the expression of STAT3 downstream target genes. Figures 5A–5D showed that the mRNA and protein expression levels of cyclin D1 and Mcl-1 decreased after Atn treatment in MDA-MB-231 and MDA-MB-468 cell lines. STAT3-Y705 phosphorylation has two protonation states, Y1P and Y2P [31]. To investigate the interaction contribution spectrum of binding free energy on DNA and STAT3-WT or STAT3-Y705 phosphorylation complex, we calculated the absolute binding free energy values for the two systems by using MM-GBSA method. The corresponding total binding free energy values of STAT3-Y1P-DNA and STAT3-Y2P-DNA complexes are −280.26 and −368.38 kcal/mol, respectively (Table 2); these results implied that the protein STAT3-Y2P exhibited higher binding affinity to DNA, and the binding free energy of the STAT3-Y2P to DNA increased by 88.12 kcal/mol compared with that of the STAT3-Y1P-DNA complex. We concluded that Y2P is the most favorable protonation state.

**Table 2:** Binding Free Energies (kcal/mol) in each system

System	ΔE_ele_	ΔE_VDW_	ΔG_GAS_	ΔG_GA_	ΔG_GB_	ΔGB_SOL_	ΔGB_ele_	ΔG_bind_	-TΔS	ΔΔG_TOT_
STAT3- Y1P-DNA	−693.95	−37.08	−731.03	−5.98	427.02	421.04	−266.93	−309.99	29.73	−280.26
STAT3- Y2P-DNA	−1116.72	−40.42	−1157.13	−6.78	761.72	754.94	−355.00	−402.19	33.81	−368.38
STAT3- WT-DNA	−364.73	−18.50	−383.23	−2.05	307.30	305.25	−57.43	−77.98	37.19	−40.79
STAT3- Y2P-DNA-Atn	−989.83	−32.35	−1022.18	−5.11	803.49	798.37	−186.34	−223.81	34.54	−198.27

Average contributions to the binding free energies in dynamic systems calculate over the last 50 ns of each system MD trajectory corresponding to equilibrium. ΔE_ele_ and ΔE_vdw_ are the molecular mechanics electrostatic and van der Waals contributions; ΔG_SA_ is the polar component of the solvation free energy, whereas ΔG_GB_ is the nonpolar component. TΔS is the entropic contribution. ΔG_bind_ is the total binding energy (calculated as the average of the individual ΔE_ele_+ ΔE_vdw_ +ΔG_SA_ +ΔG_GB_ contributions for each MD snapshot); ΔG_bind_ = ΔE_ele_ + ΔE_vdw_ + ΔG_SA_ + ΔG_GB;_ ΔΔG_TOT_ = ΔE_ele_ + ΔE_vdw_ + ΔG_SA_ + ΔG_GB_ - TΔS.

For comparison, the two average conformations (STAT3-WT-DNA and STAT3-Y2P-DNA) of the last 50 ns of the MDS trajectories were superimposed on the crystal structure (Figure 5E), which significantly differed from each other. STAT3-Y705P bound to the T-rich minor groove of DNA in a closely chimeric conformation (Figure 5E), which cannot be found in STAT3-WT-DNA average conformation. As observed from the STAT3-WT-DNA average conformation, DNA escaped from the protein WT-STAT3. Analysis of binding free energy decomposition further showed that DNA could bind to STAT3-Y705P through residues Leu382 to Arg469 with high affinity (Figures 5F and 5G). DNA could not bind to WT-STAT3 because of inactive conformation. As listed in Table 2, a lower affinity was observed between protein WT-STAT3 and DNA. We concluded that Y705P plays a key role in DNA binding to STAT3, which is blocked by Atn binding to the SH2 domain of STAT3-Y705P.

### Hydrogen bond analysis in binding modes

Atn inhibits constitutively active STAT3 (Y705) in human TNBC cells. As such, we analyzed hydrogen binding modes between DNA and protein pSTAT3 in all atomic levels. The STAT3/Y705P-DNA hydrogen bonds were analyzed (Figure 5H, Table 3). Seven hydrogen bonds were identified between the carbon atoms in sugar moieties, phosphate and oxygen atoms of the DNA backbone and STAT3-b interfaces; these bonds are the following: Arg414-1007DT, Thr341-1007DT, Arg423-1006DT, Ser465-1006DT Arg382-1006DT Gln469-1005DT, and Asn466-1007DT. STAT3-b exclusively formed hydrogen bonds with the DNA sugar-phosphate oxygen backbone. Protein residues are always hydrogen bond acceptors, and the DNA bases are hydrogen bond donors. In the system STAT3-WT-DNA, fewer hydrogen bonds are formed between STAT3-b and DNA; the occupancy period of these interactions is generally longer compared with the numerous interactions formed by STAT3-b. This observation is caused by the higher mobility of the DNA-binding domain of STAT3-b, which allows hydrogen bond interacting residues (Arg414, Thr341, Arg423, Ser465, Arg382, Gln469, and Asn466) to move far from the DNA T-rich groove; by contrast, the interface with the STAT3-b is less variable as the simulation time progresses.

**Table 3:** Percentage of Hydrogen Bonds (> 30%) MD Simulations between the DNA and the residues in STAT3- Y2P-DNA

System	donor	acceptor	Occupancy (%)	distance (Å)	angle (°)
STAT3-PY705-DNA	1007DT@O1P	Arg 414@HH21-NH2	99.40	2.885	23.49
	1007DT @O2P	Thr 341@HG1-OG1	97.00	2.681	15.87
	1006DT @O1P	Arg 423@HH11-NH1	100.00	2.792	24.24
	1006DT @O2P	Ser 465@HG-OG	98.60	2.657	15.59
	1006DT @O1P	Arg 382 @HH21-NH2	59.94	2.859	30.20
	1005DT @O1P	Gln 469@HE21-NE2	94.71	2.829	16.30
	1005DT @O2P	Asn 466@HD22 -ND2	88.01	2.900	25.01

For the dynamic system, the cut-off used for hydrogen bond distance and angle is 3.5 Å and 120°, respectively. The percentage of hydrogen bond occupancy refers to hydrogen bonds formed during the MD simulations. In addition, each hydrogen bond distance is measured between the heavy atom of donor and the heavy atom of acceptor.

Overall, we conclude that Atn inhibited TNBC growth and invasion and induced cancer cell apoptosis mainly through the inhibition of the STAT3 signal pathway (Figure 5I).

### Atn synergistically augments Taxotere^®^-induced cytotoxicity and apoptotic effects in TNBC cells

**Figure 6:**
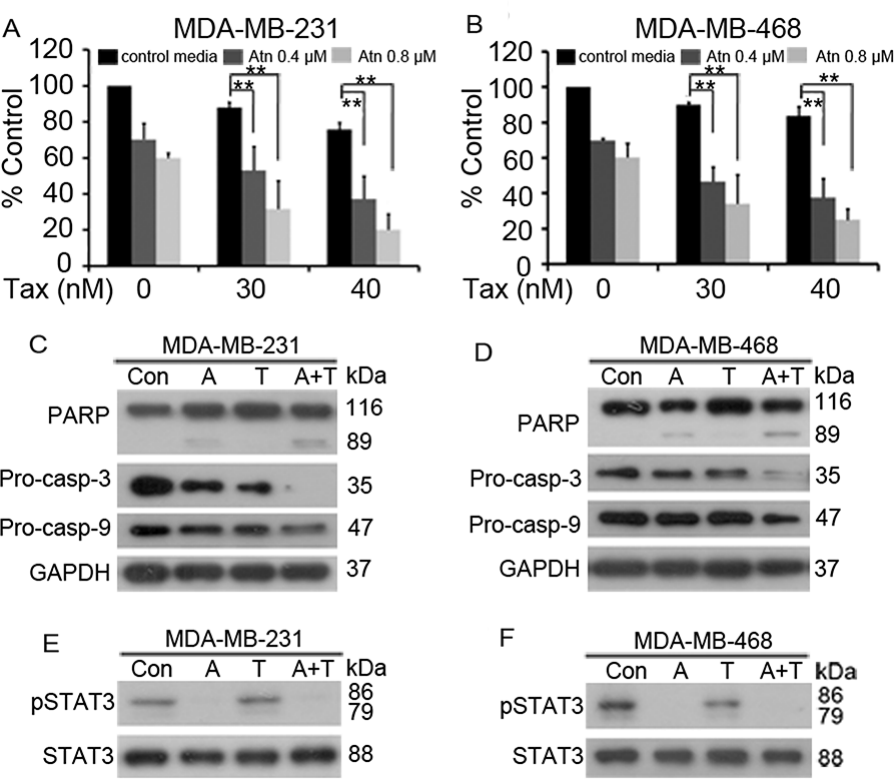
Atn enhances cytotoxicity of Taxotere^®^ to TNBC cells (**A** and **B**): MDA-MB-231 and MDA-MB-468 cells were treated for 24 h with Taxotere^®^ (Tax, 0, 30, 40 nM) in the presence of Atn at 0, 0.4, 0.8 μM. MTT assay was used to test the proliferation of cells. *P < 0.05, **P < 0.001. (**C–F**): MDA-MB-231 and MDA-MB-468 cells were cultured with control media (Con), Atn (A, 0.8 μM), Tax (T, 30 nM), or Atn (0.8 μM) plus Tax (30 nM) (A+T) for 24 h. Cells were then lysed and subjected to Western blot using indicated antibodies.

STAT3 plays a crucial role in promoting TNBC cell survival, anti-apoptosis, and drug resistance; thus, we hypothesized that the inhibition of STAT3 activity by Atn can enhance the cytotoxicity of the conventional therapeutic agent Taxotere^®^ (Tax). MDA-MB-231 and MDA-MB-468 cells were treated with Tax for 24 h in the presence of Atn (0, 0.4, and 0.8 μM) and then analyzed through MTT assay. Our results showed that Atn significantly enhanced the cytotoxicity of Tax (Figures 6A and 6B). We analyzed the CI value by using the formula assisted with CalcuSyn software (Version 2.1) and found that CI values were less than 1 (Table 4). This finding indicated that Atn and Tax synergistically inhibited TNBC cells. To gain insights into the molecular mechanisms underlying the combined use of Atn and Tax, we investigated the apoptosis pathway in MDA-MB-231 and MDA-MB-468 cells co-incubated with Atn (0.8 μM)/Tax (30 nM) combinatory regimen for 24 h. Treatment with Atn/Tax combination for 24 h induced enhanced activation of caspase-9 and −3, cleavage of caspase-3 substrate PARP, and decreased the level of STAT3 phosphorylation; this finding indicated the presence of a significant apoptosis activation (Figures 6C–6F).

**Table 4:** Atn and Tax combination index (CI) values

MDA-MB-231			MDA-MB-468		
Atn (μM)	Tax (nM)	CI	Atn (μM)	Tax (nM)	CI
0.4	30	0.589	0.4	30	0.449
0.8	40	0.192	0.8	40	0.254

MDA-MB-231 and MDA-MB-468 cells were treated with Atn and Tax combinedly or alone with indicated concentrations for 24 h, the cytotoxicity was analyzed by MTT assay, and the CI values were calculated using CalcuSyn softare (Version 2.1).

### Effect of Atn on breast cancer xenograft tumor growth *in vivo*

**Figure 7:**
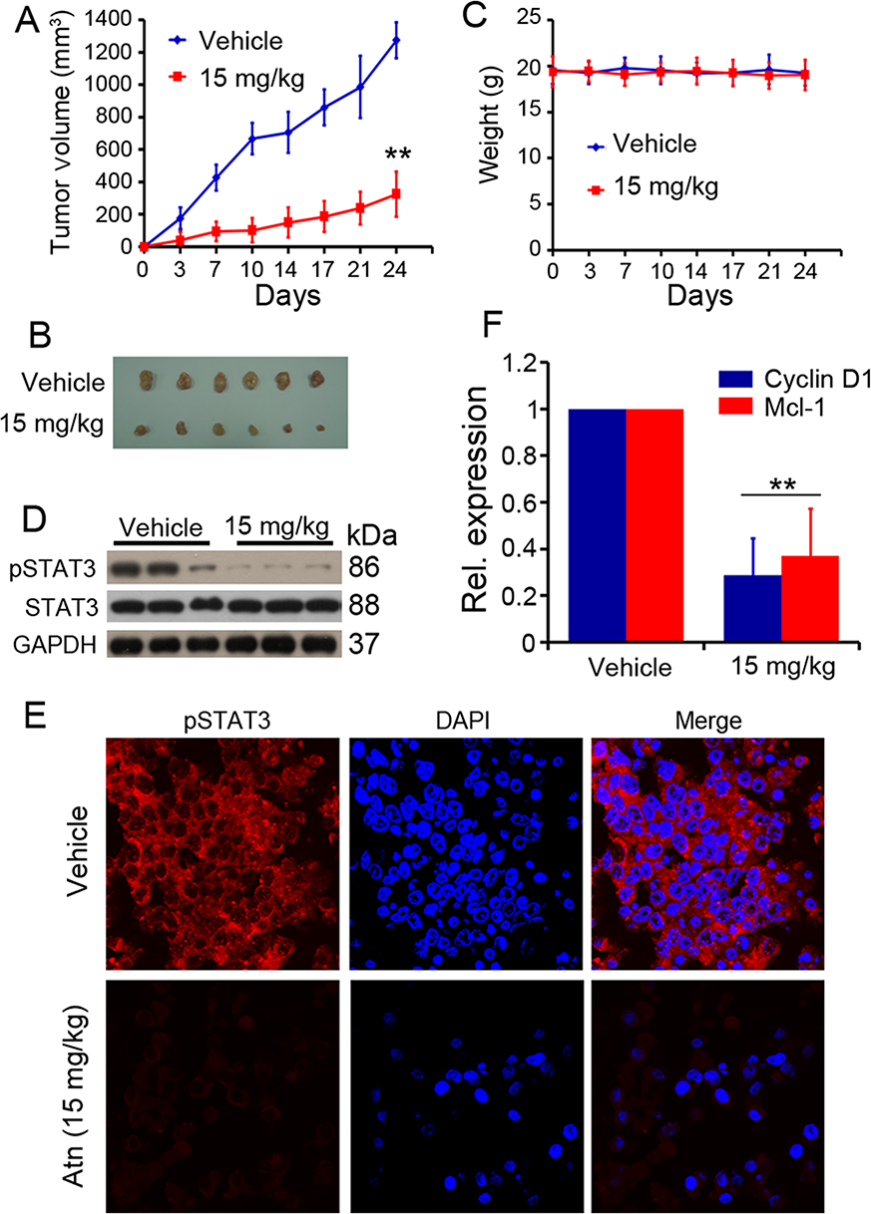
*In vivo* therapeutic efficacy of Atn on human TNBC murine models (**A**) Atn significantly inhibited MDA-MB-231 tumor growth. *P < 0.05 vs vehicle. (**B**) Images of xenograft tumors obtained from mice. (**C**) Treatment with Atn did not alter animal body weight. (**D**) pSTAT3 expression level of tumor sample lysates were analyzed by Western blot. Vehicle (Line 1–3), and Atn (Line 4–6). (**E**) pSTAT3 expression in MDA-MB-231 tumor samples was detected by tumor immunofluorescence staining. (**F**) Cyclin D1 and Mcl-1 expression in MDA-MB-231 tumor samples was detected by Real-time PCR. **P < 0.01 vs vehicle.

We tested the anti-TNBC efficacy of Atn *in vivo*. Nude mice were subcutaneously inoculated into the right flank with MDA-MB-231 cells. Tumor-bearing mice were treated with vehicle (*n* = 7) or Atn (*n* = 8) i.p. at 15 mg/kg four times a week for 4 weeks. The animals were humanely killed when their tumors reached 1.5 cm in diameter or when paralysis or a major compromise in their quality of life occurred. Figures 7A and 7B showed that Atn significantly inhibited MDA-MB-231 tumor growth (*P* < 0.01). Moreover, treatment with Atn did not reduce the body weight of mice, thereby suggesting that Atn had no obvious toxic side effects (Figure 7C). The tumor samples of mice bearing MDA-MB-231 cells were isolated. The cells were harvested, and experiments were conducted to determine whether Atn inhibits STAT3 activation *in vivo*. Remarkably, samples from Atn-treated mice showed downregulated pSTAT3 levels compared with tumor tissues from vehicle mice (Figures 7D and 7E). The expression of STAT3 downstream genes (cyclin D1 and Mcl-1) was also detected by real-time quantitative PCR, and samples from Atn-treated mice showed downregulated cyclin D1 and Mcl-1 mRNA expression levels compared with tumor tissues from vehicle mice (Figure 7F). These results demonstrated that the administration of Atn exhibits considerable potential in TNBC therapy.

## DISCUSSION

Chinese herbal medicine represents traditional medicines, such as various efficacious plants or plant extracts, which have been known to enhance healing of various diseases for thousands of years. In the field of oncology, natural small-molecule compounds from Chinese herbal medicines have been the inspiration for drug discovery. Atn is a phenylpropanoid dibenzylbutyrolactone lignan that inhibits the growth of several cancer cells, including those of the lungs, colon, liver, leukocytes, and stomach [22, 32, 33]. Hsieh [34] et al. reported that Atn induces apoptosis of MDA-MB-231 cells through the ROS/p38 MAPK pathway and epigenetic regulation; they found that Atn suppresses the proliferation of MDA-MB-231 cells with 48 h IC_50_ of 1.98 μM. However, the present study showed that the 24 h IC_50_ of Atn on MDA-MB-231cells was 0.787 μM. Atn also inhibited other breast cancer cell lines with 24 h IC_50_ values of 0.285–3.756 μM (Table 1); these cell lines included MDA-MB-468, MDA-MB-453, and MDA-MB-435S. Our results indicated that TNBC cell lines MDA-MB-231 and MDA-MB-468 were more sensitive to Atn treatment. Atn also inhibited anchorage-dependent (cell proliferation) and anchorage-independent (colony formation) growth of TNBC cells (Figure 1).

Apoptotic pathways that ultimately lead to the activation of effector caspases (casp-3, casp-2, and casp- 7) and PARP cleavage have been characterized in breast cancer [35]. Moreover, apoptosis is the main mechanism of cell death induced by chemotherapy drugs. In this study, Atn induced casp-3 and casp-9 activation, followed by PARP cleavage in two TNBC cell lines (Figure 2); thereby suggesting that Atn induces an internal caspase-dependent apoptosis in TNBC cells. In conclusion, Atn treatment showed proliferation inhibitory effect and cell apoptosis in TNBC cells, which were associated with caspase activation.

We found a different mechanism of Atn inhibiting TNSC cells in MDA-MB-231 and MDA-MB-468 cells through inhibition of STAT3 phosphorylation activation. STAT3 is an attractive cancer drug target molecule because of its over-expression and constituted activation in numerous cancers [36, 37]. Constitutive activation of STAT3 in malignance is derived from various mechanisms, such as a lack of negative regulation of upstream kinases, upstream positive signaling from mutated kinases, or other feedback inhibitors. This continuously activated state of STAT3 pathway promotes tumor cell growth, migration, invasion, angiogenesis, metastasis, and drug resistance, but inhibits apoptosis. New evidences also demonstrated that STAT3 promoted cancer progression through participation in EMT [38], tumor microenvironment regulation [39] and cancer stem cell self-renewal and differentiation [40]. STAT3 abnormal activity accelerates IL-6, HIF1α and VEGF, specifically in the tumor microenvironment. In the loop, IL-6 is involved in cancer progressions, and the elevation in serum predicts poor prognosis in certain cancers, including breast cancer [26, 27]. In gefitinib-resistant non-small-cell lung cancer, IL-6R/JAK1/STAT3 signaling activation leads to de novo resistance to irreversible EGFR inhibitors [41]. In human liver cancer cells, inactivated STAT3 causes the inhibition of IL-6-mediated anti-apoptotic activity. EGFR over-activation is correlated with breast cancer progression through activation of STATs 1 and 3. In an investigation of primary breast carcinomas and normal tissues via immunohistochemical assay, nuclear STAT3 is significantly correlated with EGFR expression in breast cancers [42]. In conclusion, both IL-6 and EGFR promote breast cancer through STAT3 abnormal activation and predict a poor prognosis. In this study, we presented that Atn inhibits IL-6 and EGF-induced STAT3 phosphorylation, but with no effects on EGFR phosphorylation (Figures 3D–3G).

We used a combined computational and experimental approach to investigate the molecular direct binding, as well as the mode of interaction of Atn with STAT3. Computational docking and MDS showed that Atn exhibited high-affinity interaction with the SH2 domain of STAT3 (Figure 4A). The overlap of the Atn binding site with those of other STAT3 inhibitors was not observed. Most STAT3 inhibitors are predicted to bind either to the hydrophilic site, lined by the side chains of the Lys591, Arg609, Ser611, and Ser613 residues, or to the partially hydrophobic region composed of the Lys592, Arg595, Ile597, and Ile634 residues [43]. Atn interacted with a consistent number of residues (Arg688, Pro689, His694, and Pro695) in the SH2 domain (Figure 4B). Furthermore, Bio-Atn/streptavidin agarose pull down experiment demonstrated the direct interaction of Atn/STAT3 (Figure 4D and 4E). Moreover, to confirm which residues were critical for the Atn/STAT3 interaction, site-directed mutagenesis on STAT3 and then immunoprecipitation and S. agarose affinity assay demonstrated that Arg688, Pro689, and Pro695 are critical for Atn binding (Figure 4F). Our results demonstrated that Atn binds to the SH2 domain and interferes directly with STAT3 activation and signaling. Higher affinity with STAT3 likely leads to higher potency in cellular assays. Moreover, Atn induced the mobility of the DNA-binding domain of STAT3-b, resulting in far location of hydrogen bond interacting residues (Arg414, Thr341, Arg423, Ser465, Arg382, Gln469, and Asn466) from the DNA T-rich groove. Therefore, Atn inhibits STAT3 downstream target gene expression in cellular assays. In summary, with the aid of structure-based virtual screening, we found that Atn can inhibit STAT3 by occupy its SH2 domain. Furthermore, Atn inhibited the DNA-binding activity of STAT3 via inhibiting constitutively active STAT3 (Y705). Hydrogen bond binding modes indicated that Atn inhibits STAT3 binding to genomic DNA by disrupting the hydrogen bond binding between DNA and STAT3 (Figure 5).

Atn also showed enhanced cytotoxicity to TNBC cells when combined with the conventional agent Tax, a drug that is currently in clinical trials for the treatment of solid malignancies (Figure 6). We subcutaneously injected MDA-MB-231 cells into nude mice and treated the animals with vehicle control and Atn. After 4 weeks of treatment, Atn significantly repressed tumor growth (Figures 7A and 7B). We euthanized the tumor-bearing mice, collected the tumors, extracted the proteins, and found that Atn downregulated the STAT3 phosphorylation (Figure 7D). The fluorescent immunohistochemistry also confirmed the inhibition (Figure 7E).

In summary, STAT3 is a novel target of Atn action in TNBC cells. This interaction may provide a significant benefit to TNBC patients, particularly in combination with other conventional agents, because STAT3 has diverse functions and is almost universally activated in TNBC cells.

## MATERIALS AND METHODS

### Reagents

Arctigenin (Atn) with a purity of up to 98% was purchased from Shanghai Yuanye Bio-Technology Co., Ltd. Atn was dissolved in DMSO (Sigma-Aldrich, U.S.A.) at a stock solution of 50 mM and stored at −20°C. Biotinylated Atn was synthesized by Boshixing Synthetic Technologies, Inc. (Shenzhen, China): biotin was condensed with 2-aminoethylthiol using dicyclohexylcarbodiimide to afford the corresponding amide, the thiol group of which was added to the enone moiety of Atn to provide the desired biotinated Atn. The identity of the compound was verifed using nuclear magnetic resonance, and the purity of biotin-Atn was determined by HPLC. Commercial Taxotere^®^ was from Sanofi-Aventis Pharma Dagenham, U.K. (Surrey, UK).

### Cell culture

Human breast cancer cell lines MCF-7, MDA-MB-231, SK-BR3, MDA-MB-468, MDA-MB-453, and MDA-MB-435S and Non-tumorigenic MCF-10A human mammary epithelial cells were obtained from American Type Culture Collection (ATCC). MCF-7 and SK-BR3 cells were maintained in Dulbecco's modified Eagle's medium (DMEM) (Gibco) supplemented with 10% fetal bovine serum (FBS, Hyclone) and antibiotics and incubated in a humidified atmosphere with 5% CO_2_ at 37°C. MDA-MB-231, MDA-MB-468, MDA-MB-453, and MDA-MB-435S cells were maintained in Leibovitz's L-15 (Gibco) supplemented with 10% FBS and Atnibiotics and incubated in a humidified atmosphere without CO_2_ at 37°C. MCF-10A cells were maintained in DMEM/F12 medium containing 5% horse serum (HS), insulin (10 mg/ml), epidermal growth factor (EGF, 20 ng/ml), choleratoxin (100 mg/ml), hydrocortisone (0.5 mg/ml), penicillin (50 U/ml), and streptomycin (50 U/ml), and incubated in a humidified atmosphere with CO_2_ at 37°C.

### Cell proliferation and cell viability

Cells were seeded into 96-well plate and pre-cultured for 24 h, then treated with Atn for 24 h or 48 h. Cell proliferation was determined by MTT assay. Cell viability was estimated by trypan blue dye exclusion.

### Soft-agar colony formation assay

Cells were suspended in 1 ml of DMEM or L-15 containing 0.3% low-melting-point agarose (Amresco, USA) and 10% FBS, and plated on a bottom layer containing 0.6% agarose and 10% FBS in 6-well plate in triplicate. After 2 weeks, plates were stained with 0.2% gentian violet and the colonies were counted under light microscope [6].

### Invasion assay

An invasion assay was carried out using 24-well plate (Corning). A polyvinyl-pyrrolidone-free polycarbonate filter (8 μm pore size) (Corning) was coated with matrigel (BD). The lower chamber was filled with medium containing 20% FBS as chemoattractant. The coated filter and upper chamber were laid over the lower chamber. Cells (1 × 10^4^ cells/well) were pre-incubated with Atn for 30 min at room temperature, and then cell suspension containing Atn was seeded onto the upper chamber wells. After incubation for 20 h at 37°C, the filter was fixed and stained with 2% ethanol containing 0.2% crystal violet (15 min). After being dried, the stained cells were enumerated under light microscope at 10 × objective. For quantification, the invaded stained cells on the other side of the membrane were extracted with 33% acetic acid. The absorbance of the eluted stain was determined at 570 nm.

### Wound healing assay

Cells (4 × 10^5^ cells/2 ml) were seeded in a 6-well plate and incubated at 37°C until 90% to 100% confluence. After the confluent cells were scratched with a 200 μl pipet tip, followed by washing with PBS, and then treated with Atn in a complete medium. After 24 h of incubation, the cells were fixed and stained with 2% ethanol containing 0.2% crystal violet powder (15 min), and randomly chosen fields were photographed under a light microscope at 4× objective. The number of cells migrated into the scratched area was calculated.

### Western blot

Cell pellets were lysed in RIPA buffer containing 50 mM Tris pH 8.0, 150 mM NaCl, 0.1% SDS, 0.5% deoxycholate, 1% NP-40, 1 mM DTT, 1 mM NaF, 1 mM sodium vanadate, 1 mM PMSF (Sigma), and 1% protease inhibitors cocktail (Merck). Protein extracts were quantitated and loaded on 8% to 12% sodium dodecyl sulfate polyacrylamide gel, electrophoresed, and transferred onto a PVDF membrane (Millipore). The membrane was incubated with primary antibody, washed, and incubated with horseradish peroxidase (HRP)-conjugated secondary antibody (Pierce). Detection was performed by using a chemiluminescent Western detection kit (Cell Signaling Technology). The antibodies used were Anti-caspase-3, Anti-caspase-9, Anti-PARP, Anti-pSTAT3 (Y705), Anti-Jak2, Anti-pJak2 (Y1007/Y1008), Anti-pSTAT3 (S727), Anti-STAT1, Anti-pSTAT1 (Y701), Anti-STAT5, and Anti-pSTAT5 (Y694) (Cell Signaling Technology), Anti-Lamin B, Anti-Cyclin D1 (Santa Cruz Biotechnology), Anti-Mcl-1, Anti-STAT3 (Proteintech), and Anti-GAPDH (Abmart) antibodies.

### Isolation of nuclear and cytoplasmic fractionation

Nuclear and cytoplasmic extracts were prepared with the NE-PER Nuclear and Cytoplasmic Extraction Kit (Thermo Scientific). The purity of nuclear and cytoplasmic extracts was assessed by Western blot with anti-Lamin B and anti-GAPDH antibodies, respectively.

### Real-time quantitative PCR

Expression of genes was examined by real-time quantitative PCR normalized to expression of GAPDH. Total RNA was extracted from cell lines or patients' cells using Trizol reagent (Invitrogen). Quantitative real-time PCR was performed using SYBR Premix Ex Taq (Perfect Real Time) (TaKaRa) according to the manufacturer's instruction [44]. For real-time PCR, we used Cyclin D1 gene forward primer 5′-CCGTCCATGCGGAAGATC-3′, Cyclin D1 gene reverse primer 5′-GTCACACTT GATCACTCTGG-3′; Mcl-1 gene forward primer 5′-CACG AGACGGTCTTCCAAGGCATGCT-3′; Mcl-1 gene reverse primer 5′-CTAGGTTGCTAGGGTGCAACTCTA GGA-3′; GAPDH forward primer 5′- TGTTGCCATCAATG ACCCCTT-3′, GAPDH reverse primer 5′- CTCCACG ACGTACTCAGCG-3′.

### Computational studies

The crystal structures of STAT3 protein was obtained from the available pdb file 1BG1 in the Protein Data Bank repository. The chemical structure of Atn is shown in Figure 1A. For the sake of getting the most stable conformation and the electrostatic potential distributions, we calculated single point energies at the HF/6-31G (d,p) level by Gaussian 09 program [45]. All docking experiments were performed with Autodock 4.2. For the AutoDock 4.2 calculation, the grid maps employ a grid module with 70 × 70 × 70 points of 0.375Å spacing. We employed Lamarckian genetic algorithm for the docking calculation of the protein and inhibitor, as the following options: population size of individuals 150, a maximum number of 25 million energy evaluations, a maximum number of generations of 27000, a crossover rate of 0.8, a mutation rate of 0.02, and independent docking runs of 50 were set up. The docked conformations were clustered using a tolerance of 2 Å root-mean-square deviations and evaluated for the final docking structure according to the binding free energy. For MD simulations, the complex protein was performed by using the AMBER12 program. The AMBER ff99SB force field parameters were adopted for the MD simulations. The binding free energy, ΔGbind, between drug/DNA and the protein was estimated resorting to the MM/PBSA (Molecular Mechanics/Poisson-Boltzmann Surface Area) approach [46]. According to this well-validated methodology, the binding free energy was obtained as the sum of the interaction energy between the receptor and the ligand (ΔEMM), the solvation free energy (ΔGsol), and the conformational entropy contribution (−TΔS), averaged over a series of snapshots from the corresponding MDS trajectories [47].

### Immunoprecipitation and streptavidin agarose affinity assay

Cell pellets were lysed and the supernatant collection was incubated with indicated antibodies overnight at 4°C, after which protein A/G Plus beads (Santa Cruz Biotechnology) were added and incubated at 4°C for 4 h. The beads were washed 4 times in NETN buffer (1% NP- 40, 2 mM EDTA, 40 mM Tris-HCl, 137 mM NaCl, pH 7.4), resuspended in SDS-PAGE loading buffer and boiled for 5 min. For S. agarose affinity assay, cells upon Bio-Atn were lysed, the lysates were incubated with streptavidin agarose, washed and boiled in SDS-PAGE loading buffer. For Atn competition, the cell lysates were pretreated with Atn (10 μM) for 1 h, followed by 50 μM Bio-Atn treatment for 3 h at 4°C, and S. agarose affinity assay were performed. Western blot assays were performed as described.

### Drug combination assay

Drug combination is widely used in cancer treatment to achieve synergistic therapeutic effect and overcomes drug resistance in clinic. To estimate the effect of Atn and Taxotere^®^ (Tax) combination, the combination index (CI) was calculated by the Chou-Talalay equation. MDA-MB-231 and MDA-MB-468 cells were seeded in 96-well plates. Drugs were added alone or together at indicated concentration. The inhibition effect was measured by MTT assay as mentioned above. The formula of CI = (D)Atn/(Dx)Atn+ (D)Tax/(Dx)Tax ((D)Atn and (D)Tax: the doses of compounds Atn and Tax, respectively, necessary to produce the same effect in combination. Dx: the dose of one compound alone required producing an effect). With this formula and assistance of CalcuSyn software (Version 2.1), the combined effects of the two compounds can be assessed as followes: CI < 1 indicates synergism; CI = 1 indicates additive effect; and CI > 1 indicates antagonism.

### Human breast cancer xenograft experiments

Female nude immunodeficient mice (nu/nu), 6–8 weeks old, were purchased from Hunan SJA Laboratory Animal Co., Ltd., and maintained and monitored in a specific pathogen-free environment. All animal studies were conducted according to protocols approved by the Hubei University of Medicine Animal Care and Use Committee, complying with the rules of Regulations for the Administration of Affairs Concerning Experimental Animals (Approved by the State Council of China). The mice were injected subcutaneously with human breast cancer MDA-MB-231 cells (6 × 10^6^) suspended in 100 μl L-15 media into the right flank of each mouse. Treatments were started when the tumors reached a palpable size. Mice were randomly divided into two groups and treated 4 times per week. The control group (*n* = 7) received vehicle, while another group (*n* = 8) received intraperitoneally (i.p.) injection of Atn (15 mg/kg). Caliper measurements of the longest perpendicular tumor diameters were performed twice a week to estimate the tumor volume, using the following formula: 4π/3 × (width/2)^2^ × (length/2), representing the 3-dimensional volume of an ellipse. Animals were sacrificed when tumors reached 1.5 cm or if the mice appeared moribund to prevent unnecessary morbidity to the mice. At the time of the animals' death, tumors were excised; cells were lyzed for Real-time quantitative PCR, Western blot, or immunofluorescence.

### Tumor immunofluorescence staining

Surgically excised tumors were cryosectioned to 7 μm thick sections. Then the frozen sections were thawed and fixed with 4% paraformaldehyde for 30 min. After blocking with 3% BSA/0.2% Triton X-100 in PBS for 1 h, sections were incubated with anti-pSTAT3 antibody at 4°C overnight. Cy3 conjugated donkey anti-rabbit IgG antibody was used as the secondary antibody. For visualization of cell nucleus, DAPI was used. Sections were observed by Olympus confocal laser scanning microscope.

### Statistical analysis

All experiments were repeated at least three times and the data were presented as the mean ± SD unless noted otherwise. Differences between data groups were evaluated for significance using Student t -test of unpaired data or one-way analysis of variance and Bonferroni post -test. *P* values less than 0.05 indicate statistical significance.

## SUPPLEMENTARY MATERIALS



## References

[1] Siegel RL, Miller KD, Jemal A (2015). Cancer statistics, 2015. Cancer J Clin.

[2] Stevens KN, Vachon CM, Couch FJ (2013). Genetic susceptibility to triple-negative breast cancer. Cancer Res.

[3] Deng XS, Wang S, Deng A, Liu B, Edgerton SM, Lind SE, Wahdan-Alaswad R, Thor AD (2012). Metformin targets Stat3 to inhibit cell growth and induce apoptosis in triple-negative breast cancers. Cell Cycle.

[4] D'Amato NC, Rogers TJ, Gordon MA, Greene LI, Cochrane DR, Spoelstra NS, Nemkov TG, D'Alessandro A, Hansen KC, Richer JK (2015). A TDO2-AhR Signaling Axis Facilitates Anoikis Resistance and Metastasis in Triple-Negative Breast Cancer. Cancer Res.

[5] Goodwin CM, Rossanese OW, Olejniczak ET, Fesik SW (2015). Myeloid cell leukemia-1 is an important apoptotic survival factor in triple-negative breast cancer. Cell Death Differ.

[6] Cao W, Liu Y, Zhang R, Zhang B, Wang T, Zhu X, Mei L, Chen H, Zhang H, Ming P, Huang L (2015). Homoharringtonine induces apoptosis and inhibits STAT3 via IL-6/JAK1/STAT3 signal pathway in Gefitinib-resistant lung cancer cells. SciRep.

[7] Crescenzo R, Abate F, Lasorsa E, Tabbo' F, Gaudiano M, Chiesa N, Di GF, Spaccarotella E, Barbarossa L, Ercole E, Todaro M, Boi M, Acquaviva A (2015). Convergent mutations and kinase fusions lead to oncogenic STAT3 activation in anaplastic large cell lymphoma. Cancer Cell.

[8] Amin HM, McDonnell TJ, Ma Y, Lin Q, Fujio Y, Kunisada K, Leventaki V, Das P, Rassidakis GZ, Cutler C, Medeiros LJ, Lai R (2004). Selective inhibition of STAT3 induces apoptosis and G(1) cell cycle arrest in ALK-positive anaplastic large cell lymphoma. Oncogene.

[9] Chun J, Li RJ, Cheng MS, Kim YS (2015). Alantolactone selectively suppresses STAT3 activation and exhibits potent anticancer activity in MDA-MB-231 cells. Cancer Lett.

[10] Couto JP, Daly L, Almeida A, Knauf JA, Fagin JA, Sobrinho-Simoes M, Lima J, Maximo V, Soares P, Lyden D, Bromberg JF (2012). STAT3 negatively regulates thyroid tumorigenesis. Proc Natl Acad Sci U S A.

[11] Garcia R, Bowman TL, Niu G, Yu H, Minton S, Muro-Cacho CA, Cox CE, Falcone R, Fairclough R, Parsons S (2001). Constitutive activation of Stat3 by the Src and JAK tyrosine kinases participates in growth regulation of human breast carcinoma cells. Oncogene.

[12] Fujita DJ, Ethier SP, Jove R (1997). Constitutive Activation of Stat3 in Fibroblasts Transformed by Diverse Oncoproteins and in Breast Carcinoma Cells'. Cell Growth Differ.

[13] Pilati C, Amessou M, Bihl MP, Balabaud C, Van Nhieu JT, Paradis V, Nault JC, Izard T, Bioulac-Sage P, Couchy G (2011). Somatic mutations activating STAT3 in human inflammatory hepatocellular adenomas. The Journal of experimental medicine.

[14] Zhang F, Li C, Halfter H, Liu J (2003). Delineating an oncostatin M-activated STAT3 signaling pathway that coordinates the expression of genes involved in cell cycle regulation and extracellular matrix deposition of MCF-7 cells. Oncogene.

[15] Opdam FJ, Kamp M, de BR, Roos E (2004). Jak kinase activity is required for lymphoma invasion and metastasis. Oncogene.

[16] Zhao X, Sun X, Li XL (2012). Expression and clinical significance of STAT3, P-STAT3, and VEGF-C in small cell lung cancer. Asian PacJCancer Prev.

[17] Jiang R, Jin Z, Liu Z, Sun L, Wang L, Li K (2011). Correlation of activated STAT3 expression with clinicopathologic features in lung adenocarcinoma and squamous cell carcinoma. MolDiagnTher.

[18] Venturutti L, Romero LV, Urtreger AJ, Chervo MF, Cordo Russo RI, Mercogliano MF, Inurrigarro G, Pereyra MG, Proietti CJ, Izzo F, Diaz Flaque MC, Sundblad V, Roa JC (2015). Stat3 regulates ErbB-2 expression and co-opts ErbB-2 nuclear function to induce miR-21 expression, PDCD4 downregulation and breast cancer metastasis. Oncogene.

[19] Cho MK, Park JW, Jang YP, Kim YC, Kim SG (2002). Potent inhibition of lipopolysaccharide-inducible nitric oxide synthase expression by dibenzylbutyrolactone lignans through inhibition of I-kappaBalpha phosphorylation and of p65 nuclear translocation in macrophages. IntImmunopharmacol.

[20] de Almeida AB, Sanchez-Hidalgo M, Martin AR, Luiz-Ferreira A, Trigo JR, Vilegas W, dos Santos LC, Souza-Brito AR, de la Lastra CA (2013). Anti-inflammatory intestinal activity of Arctium lappa L. (Asteraceae) in TNBS colitis model. JEthnopharmacol.

[21] Lee YJ, Choi DH, Cho GH, Kim JS, Kang DG, Lee HS (2012). Arctium lappa ameliorates endothelial dysfunction in rats fed with high fat/cholesterol diets. BMCComplement AlternMed.

[22] Yao X, Zhu F, Zhao Z, Liu C, Luo L, Yin Z (2011). Arctigenin enhances chemosensitivity of cancer cells to cisplatin through inhibition of the STAT3 signaling pathway. JCell Biochem.

[23] Zhang X, Blaskovich MA, Forinash KD, Sebti SM (2014). Withacnistin inhibits recruitment of STAT3 and STAT5 to growth factor and cytokine receptors and induces regression of breast tumours. BrJCancer.

[24] Sanchez-Lopez E, Flashner-Abramson E, Shalapour S, Zhong Z, Taniguchi K, Levitzki A, Karin M (2015). Targeting colorectal cancer via its microenvironment by inhibiting IGF-1 receptor-insulin receptor substrate and STAT3 signaling. Oncogene.

[25] Rebouissou S, Amessou M, Couchy G, Poussin K, Imbeaud S, Pilati C, Izard T, Balabaud C, Bioulac-Sage P, Zucman-Rossi J (2009). Frequent in-frame somatic deletions activate gp130 in inflammatory hepatocellular tumours. Nature.

[26] Bachelot T, Ray-Coquard I, Menetrier-Caux C, Rastkha M, Duc A, Blay JY (2003). Prognostic value of serum levels of interleukin 6 and of serum and plasma levels of vascular endothelial growth factor in hormone-refractory metastatic breast cancer patients. British journal of cancer.

[27] Hong DS, Angelo LS, Kurzrock R (2007). Interleukin-6 and its receptor in cancer. Cancer.

[28] Zhong S, Yin H, Liao Y, Yao F, Li Q, Zhang J, Jiao H, Zhao Y, Xu D, Liu S, Song H, Gao Y, Liu J (2015). Lung Tumor Suppressor GPRC5A Binds EGFR, Restrains Its Effector Signaling. Cancer Res.

[29] Berclaz G, Altermatt HJ, Rohrbach V, Siragusa A, Dreher E, Smith PD (2001). EGFR dependent expression of STAT3 (but not STAT1) in breast cancer. International journal of oncology.

[30] Gao SP, Mark KG, Leslie K, Pao W, Motoi N, Gerald WL, Travis WD, Bornmann W, Veach D, Clarkson B, Bromberg JF (2007). Mutations in the EGFR kinase domain mediate STAT3 activation via IL-6 production in human lung adenocarcinomas. The Journal of Clinical Investigation.

[31] Homeyer N, Horn AH, Lanig H, Sticht H (2006). AMBER force-field parameters for phosphorylated amino acids in different protonation states: phosphoserine, phosphothreonine, phosphotyrosine, and phosphohistidine. J Mol Model.

[32] Susanti S, Iwasaki H, Inafuku M, Taira N, Oku H (2013). Mechanism of arctigenin-mediated specific cytotoxicity against human lung adenocarcinoma cell lines. Phytomedicine.

[33] Susanti S, Iwasaki H, Itokazu Y, Nago M, Taira N, Saitoh S, Oku H (2012). Tumor specific cytotoxicity of arctigenin isolated from herbal plant Arctium lappa L. JNatMed.

[34] Hsieh CJ, Kuo PL, Hsu YC, Huang YF, Tsai EM, Hsu YL (2014). Arctigenin, a dietary phytoestrogen, induces apoptosis of estrogen receptor-negative breast cancer cells through the ROS/p38 MAPK pathway and epigenetic regulation. Free RadicBiolMed.

[35] Ha K, Fiskus W, Choi DS, Bhaskara S, Cerchietti L, Devaraj SG, Shah B, Sharma S, Chang JC, Melnick AM, Hiebert S, Bhalla KN (2014). Histone deacetylase inhibitor treatment induces ‘BRCAness’ and synergistic lethality with PARP inhibitor and cisplatin against human triple negative breast cancer cells. Oncotarget.

[36] Frank DA (2007). STAT3 as a central mediator of neoplastic cellular transformation. Cancer Lett.

[37] Gariboldi MB, Ravizza R, Molteni R, Osella D, Gabano E, Monti E (2007). Inhibition of Stat3 increases doxorubicin sensitivity in a human metastatic breast cancer cell line. Cancer Lett.

[38] Lee Y, Jung WH, Koo JS (2015). Adipocytes can induce epithelial-mesenchymal transition in breast cancer cells. Breast cancer research and treatment.

[39] Azare J, Doane A, Leslie K, Chang Q, Berishaj M, Nnoli J, Mark K, Al-Ahmadie H, Gerald W, Hassimi M (2011). Stat3 mediates expression of autotaxin in breast cancer. PLoS One.

[40] Thakur R, Trivedi R, Rastogi N, Singh M, Mishra DP (2015). Inhibition of STAT3, FAK, Src mediated signaling reduces cancer stem cell load, tumorigenic potential and metastasis in breast cancer. Scientific reports.

[41] Kim SM, Kwon OJ, Hong YK, Kim JH, Solca F, Ha SJ, Soo RA, Christensen JG, Lee JH, Cho BC (2012). Activation of IL-6R/JAK1/STAT3 Signaling Induces De Novo Resistance to Irreversible EGFR Inhibitors in Non–Small Cell Lung Cancer with T790M Resistance Mutation. Molecular Cancer Therapeutics.

[42] Liu Y, Li P-K, Li C, Lin J (2010). Inhibition of STAT3 Signaling Blocks the Anti-apoptotic Activity of IL-6 in Human Liver Cancer Cells. Journal of Biological Chemistry.

[43] Fletcher S, Turkson J, Gunning PT (2008). Molecular approaches towards the inhibition of the signal transducer and activator of transcription 3 (Stat3) protein. ChemMedChem.

[44] Liu Y, Cao W, Zhang B, Liu YQ, Wang ZY, Wu YP, Yu XJ, Zhang XD, Ming PH, Zhou GB, Huang L (2013). The natural compound magnolol inhibits invasion and exhibits potential in human breast cancer therapy. SciRep.

[45] Tatewaki H, Koga T, Shimazaki T, Yamamoto S (2004). Quality of contracted Gaussian-type function basis sets. J Chem Phys.

[46] Durmaz V, Schmidt S, Sabri P, Piechotta C, Weber M (2013). Hands-off linear interaction energy approach to binding mode and affinity estimation of estrogens. J Chem Inf Model.

[47] Yildirim I, Kennedy SD, Stern HA, Hart JM, Kierzek R, Turner DH (2012). Revision of AMBER Torsional Parameters for RNA Improves Free Energy Predictions for Tetramer Duplexes with GC and iGiC Base Pairs. J Chem Theory Comput.

