# Supplementary material for: Arctigenin inhibits STAT3 and exhibits anticancer potential in human triple-negative breast cancer therapy
Source: Oncotarget. 2016 Nov 16;8(1):329–44. doi: 10.18632/oncotarget.13393 (PMC5352123; doi:10.18632/oncotarget.13393)
Supplement: Supplementary file 1 [file oncotarget-08-329-s001.pdf]

Arctigenin inhibits STAT3 and exhibits anticancer potential in human triple-negative breast cancer therapy

Supplementary Materials

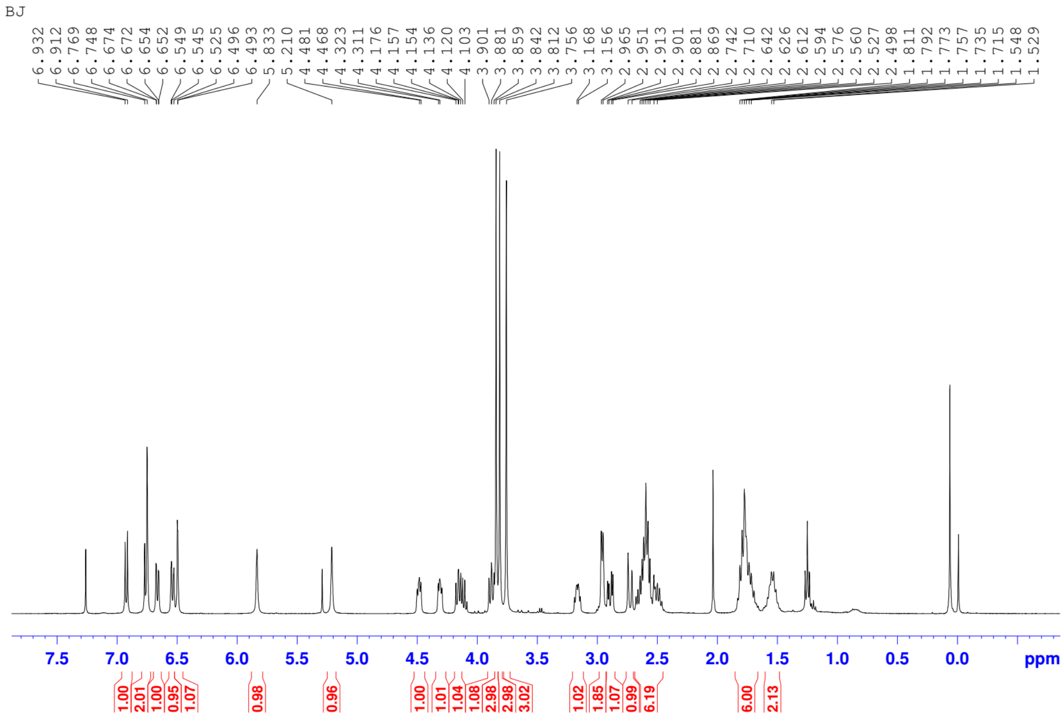

Supplementary Figure S1: Nuclear magnetic resonance analysis of Biotin-Atn.
